# Genome-wide association studies for milk production traits and persistency of first calving Holstein cattle in Türkiye

**DOI:** 10.3389/fvets.2024.1461075

**Published:** 2024-10-24

**Authors:** Metin Erdoğan, Samet Çinkaya, Bertram Brenig, Koray Çelikeloğlu, Mustafa Demirtaş, Suat Sarıibrahimoğlu, Mustafa Tekerli

**Affiliations:** ^1^Department of Veterinary Biology and Genetics, Faculty of Veterinary, Afyon Kocatepe University, Afyonkarahisar, Türkiye; ^2^Department of Animal Science, Faculty of Veterinary, Afyon Kocatepe University, Afyonkarahisar, Türkiye; ^3^Department of Molecular Biology of Livestock, Institute of Veterinary Medicine, Georg August University Göttingen, Göttingen, Germany; ^4^Kaanlar Agriculture and Livestock Farm, Çanakkale, Türkiye

**Keywords:** GWAS, candidate genes, milk production, persistency, Holstein heifers, cattle

## Abstract

The study presents a comprehensive investigation into the genetic determinants of 100-day milk yield (100DMY), 305-day milk yield (305DMY), total milk yield (TMY), and persistency using first lactation records of 374 Holstein heifers reared in a private farm at Çanakkale province of Türkiye, employing a genome-wide association study (GWAS) approach. The research underscores the substantial genetic component underlying these economically important traits through detailed descriptive statistics and heritability estimations. The estimated moderate to high heritabilities (0.32–0.54) for milk production traits suggest the feasibility of targeted genetic improvement strategies. By leveraging GWAS, the study identifies many significant and suggestively significant single nucleotide polymorphisms (SNP) associated with studied traits. Noteworthy genes have identified in this analysis include BCAS3, MALRD1, CTNND2, DOCK1, TMEM132C, NRP1, CNTNAP2, GPRIN2, PLEKHA5, GLRA1, SCN7A, HHEX, KTM2C, RAB40C, RAB11FIP3, and FXYD6. These findings provide valuable understandings of the genetic background of milk production and persistency in Holstein cattle, shedding light on specific genomic regions and candidate genes playing pivotal roles in these traits. This research contributes valuable knowledge to the field of dairy cattle genetics and informs future breeding efforts to improve milk production sustainability and efficiency in Holstein cattle populations.

## 1 Introduction

Milk is an essential natural source of valuable animal nutriments that is vital to human nutrition worldwide. Worldwide population growth necessitates the ongoing prioritization of milk yield in cattle breeding while concurrently minimizing reproductive and health issues. Oltenacu and Broom (1) highlight that regions such as Europe, North America, and Nordic Countries have already integrated the enhancement of fertility and reduction of mastitis incidence into their breeding objectives. This strategic approach underscores the importance of addressing key health and reproductive concerns in dairy cattle populations to enhance overall herd performance and welfare. By prioritizing these objectives in breeding programs, stakeholders aim to foster more resilient and productive dairy herds, ultimately contributing to sustainable and profitable dairy farming practices. Wiggans et al. (2) highlighted a significant transformation in dairy cattle breeding attributable to implementing genomic selection. The adoption of this approach has resulted in a doubling of the genetic progress rate, facilitated by the enhanced accuracy in estimating the genetic merit of young animals. The intensified utilization of genetically superior young male and female animals identified at an early age decreases the generation interval (3). Numerous researchers are dedicated to delving into animal genomes, with a particular emphasis on the multitude of genes that play significant roles in regulating production traits in dairy cattle. Candidate gene identification is crucial for the genomic background underlying the interested traits, and various methods are employed for this purpose. One of the most recent methods is the Genome-wide Association Study (GWAS), as highlighted by Taherkhani et al. (4). Several GWAS studies have been conducted with the aim of identifying quantitative trait loci (QTL), genomic regions, and/or single nucleotide polymorphisms (SNPs) associated with milk production traits in different Holstein populations (5–10). However, there is a notable lack of studies focusing on the genetic architecture of milk production traits in Holstein cattle within the specific context of Türkiye. Türkiye presents a unique environment for dairy production due to its diverse climatic regions, which can significantly influence the expression of genetic traits in cattle. These environmental variations may lead to different gene expressions affecting milk production, necessitating research that specifically addresses the genomic characteristics of Holstein cattle in Türkiye. Türkiye’s primary focus remains on enhancing milk yield, making it imperative to understand the genetic factors influencing this trait within the local context. By investigating the genetic parameters associated with milk production in Holstein cattle raised in Türkiye, this study aims to fill a critical gap in the literature and provide valuable insights for improving dairy productivity through targeted breeding programs. The objectives of this study are to estimate the genetic parameters associated with key milk production traits, including 100-day milk yield (100DMY), 305-day milk yield (305DMY), total milk yield (TMY), and persistency, and to identify novel SNPs and candidate genes linked to these traits. By employing a GWAS approach, this research looks forward to enhance our understanding of the genetic foundation of milk production in Holstein cattle under the specific conditions of Türkiye, ultimately contributing to the development of more efficient and sustainable dairy farming practices in the region.

## 2 Materials and methods

Ethical approval is not needed for this study as the animals were genotyped using routinely collected blood samples from the farm where the research was conducted.

### 2.1 Phenotyping data

The first lactation records of 374 American originated Holstein heifers raised in a private farm located in Çanakkale province were used in this study. The heifers calved between 2017 and 2021. These animals were daughters of 40 sires and 345 dams. All the data were acquired from the daily milk records collected by an automatic farm management system (GEA Farm Technologies). The cattle were fed using a standardized feeding system tailored to meet their specific needs for growth, reproduction, and milk production, and housed in well-ventilated barns with access to clean water and suitable bedding. Only the animals with lactation lengths of 200–500 days were included in the analyses. Persistency was calculated as follows:


$$\mathrm{Persistency}=\left(1-\frac{100-\mathrm{day}\;\mathrm{milk}\;\mathrm{yield}\;}{305-\mathrm{day}\;\mathrm{milk}\;\mathrm{yield}}\right)*100$$

### 2.2 Genotyping data and quality control

DNA samples of the animals were used for genotyping by Illumina BovineSNP50K BeadChip. The reference genome assembly was based on cattle genome UMD 3.1 (NCBI-http://www.ncbi.nlm.nih.gov). Before GWAS, imputation was performed using an in-home Excel VBA Add-in, which utilize the kNN algorithm. QC filtering was applied to imputed data to decrease type 1 errors. The raw data comprised 62,902 SNPs for each of the 377 cows at the end of genotyping. SNPs on sex and do not belong to any chromosomes were excluded. SNPs were excluded from subsequent analysis if they had a call rate below 95%, a minor allele frequency under 5%, a Hardy–Weinberg equilibrium (HWE) test *p*-value less than 1E-04 or were identified as duplicate SNPs. Additionally, three samples were excluded due to having over 10% missing SNP genotypes. Following data filtering, a total of 42,444 single nucleotide polymorphisms (SNPs) and 374 individual animals were retained for subsequent genome-wide association studies (GWAS) analyses. This rigorous selection process ensures that only high-quality genetic markers and a representative sample size are utilized, allowing for robust and reliable investigations into the genetic basis of the traits under study.

### 2.3 Variance components and GWAS

MINITAB software was used to obtain descriptive statistics and determine the significancy of the environmental factors for the traits. The animals with outlier records (μ ± 3 standard deviations) were discarded from the data. We estimated the variance components for each trait using average information restricted maximum likelihood (AI-REML) accommodating genomic relationship matrix by following mixed model:


y=μ+Xβ+Za+e

where y was the vector of phenotypic records; μ was the population mean related to the traits; β was the vector of significant environmental fixed effects including calving year (2017–2021) and season (winter, spring, summer, and fall); a was the vector of random animal effects; e was the random residual error; and X and Z were incidence matrices attributing phenotypic observations to fixed and additive genetic effects. The lactation length was added to the model as a covariate in the run of TMY analysis. WOMBAT (Version 07/03/2023) (11) was used in the formation of a genomic relationship matrix and estimation of variance components and multiple SNP effects with the guide of the software handbook. Genome-wide (1.17 x 10E-06, = 0.05/42444) and chromosome-wide [3.42 x 10E-05, = 0.05/(42,444/29)] Bonferroni thresholds were used for statistical significance. Additionally, suggestive threshold of <3.00 x 10E-04 was considered suggestively significant for associations. Manhattan plots to display the -log10 (*p*-value) of SNP effects and Quantile-Quantile (Q-Q) plots to compare the genome-wide distribution of the test statistics with the expected null distribution were created using the qqman package (12) version 0.1.8 in R version 4.3.0 (The R Foundation, Vienna, Austria). The median of the observed chi-squared test statistic divided by the expected median of the corresponding chi-squared distribution is the definition of genomic inflation factor (λ). According to Williams et al. (13), a genomic inflation factor (λ) close to 1 indicates no significant evidence of inflation, suggesting that the statistical analysis is not biased by population stratification or other confounding factors. In the context of genome-wide association studies (GWAS), values of λ up to 1.10 are typically deemed acceptable, as they indicate minimal inflation and thus maintain the integrity of the results.

### 2.4 Gene annotation and functional enrichment analysis

National Center for Biotechnology Information database^1^ were used to obtain nearby genes related to each SNP. Genes including or nearest to significant SNPs were considered as candidates.

Gene Ontology (GO) functional annotation and KEGG signaling pathway analysis were performed to gain deeper insights into the biological significance of the candidate regions in various biological processes, molecular functions, and cellular components. The genes located within 200 kb upstream and downstream of these SNPs were submitted to the Database for Annotation, Visualization and Integrated Discovery (DAVID) platform (14, 15). A *p*-value below 0.05 was deemed statistically significant in the enrichment analysis.

## 3 Results and discussion

The descriptive statistics for milk production traits and persistency of Holstein cattle are shown in Table 1. Additive and residual variance components are shown in Table 2.

**Table 1:** Descriptive statistics of 100DMY, 305DMY, TMY, and persistency for Holstein heifers.

Traits	n	Mean	SD	CV	Minimum	Maximum
100DMY, kg	373	3,748	549	14.65	1,929	5,342
305DMY, kg	374	11,217	1,624	14.48	6,781	15,629
TMY, kg	299	12,827	2,731	21.29	6,346	20,587
Persistency, %	366	66.36	3.10	4.67	57.22	74.18

**Table 2:** Variance components and heritability estimates for 100DMY, 305DMY, TMY, and persistency.

Traits	$\sigma_{a}^{2}$	$\sigma_{e}^{2}$	$\sigma_{P}^{2}$	$h^{2}$
100DMY	0.111 ± 0.033	0.153 ± 0.027	0.264 ± 0.021	0.42 ± 0.11
305DMY	1.381 ± 0.344	1.177 ± 0.257	2.558 ± 0.206	0.54 ± 0.11
TMY	1.595 ± 0.476	1.833 ± 0.391	3.427 ± 0.301	0.47 ± 0.12
Persistency	2.840 ± 1.049	6.069 ± 0.950	8.909 ± 0.693	0.32 ± 0.11

### 3.1 Genetic parameters

Estimated direct heritabilities for the 100DMY, 305DMY, TMY, and persistency were 0.42, 0.54, 0.47, and 0.32, respectively. These estimations were moderately high and implied a possibility for faster genetic progress. Some cows start the lactation with a high production capacity. However, inadequate energy intake in high-yielding cows during early lactation can elevate the risk of ketosis, which arises from an energy imbalance (16). According to Gustafsson et al. (16), ketotic animals exhibited lactation curves with an abnormal shape, characterized by an inverted peak during early lactation. Most of the decrease in milk yield for these animals likely occurred within the first 100 days of lactation. Moderate genetic variability for this trait may provide an opportunity to improve the starting yields of the cows and/or heifers. Utilizing both genomic relationship and pedigree-based information in heritability estimation may lead to more accurate and higher heritability estimates for 305DMY. Our study yielded a notably higher estimate (0.54) for the 305DMY. This finding contrasts with previous research by Nayeri et al. (17), Atashi et al. (6), Eiríksson et al. (18), and Zhou et al. (19), who reported higher and closer heritabilities for 305DMY, ranging from 0.37 to 0.43 in Holstein and Xinjiang Brown breeds. Conversely, several studies documented lower heritability estimates, ranging from 0.03 to 0.29, across various cattle breeds (5, 20–23). Additionally, Schmidtmann et al. (24) reported a heritability of 0.333 for the 305-day milk yield in the first lactation of German Holstein cattle. Regarding the TMY trait, our study found a moderate heritability estimate of 0.47, contrasting with a lower estimate of 0.24 reported by Eiríksson et al. (18) in Icelandic dairy cows. These variations in heritability estimates across studies underscore the influence of factors such as breed, management practices, and environmental conditions on milk production traits in cattle populations. The difference could be due to the genomic relationship matrix used in this study, breed, and statistical methods. Heifers that demonstrate lower milk production during the first 100 days of lactation, in contrast to the rest of the lactation period, typically show greater persistency. The heritability estimate for persistency was 0.32, indicating the potential for a more robust selection response. Madsen (25) and Tekerli et al. (26) underlined that the moderate yield at the beginning of lactation with moderate persistency minimizes the problems leading to reproductive or metabolic disorders. Focusing solely on milk yield during selection can lead to a significant negative energy balance in cows. This imbalance prompts cows to mobilize body fat reserves to compensate for the lack of nutrients required for milk production (27, 28). Moreover, Pereira et al. (28) cautioned against solely focusing on total milk yield in selection processes, emphasizing the importance of considering lactation persistency in dairy breeding programs. These insights highlight the need for a more holistic approach to breeding strategies, one that accounts for both milk quantity and persistency to ensure the long-term health and productivity of dairy herds. Cows with a flat curve require less concentrate feed during lactation, so we suggest keeping these animals in the herd. Therefore, an index considering milk yield, persistency, milk fat, protein and other sustainable criteria could be used for the improving of dairy cows. In Table 3, genetic and phenotypic correlations among all traits are presented. These correlations were notably strong and positive, ranging between 0.70 and 0.99 for both genetic and phenotypic measures. Strong correlations between 100DMY and 305DMY or TMY indicated that the cows starting lactation with a high yield have the genetic potential to give more milk in the remaining part of the lactation. Genetic and phenotypic correlations of persistency with 100DMY were negative and implied the cows giving most of the lactation milk yield in the first 100 days could have a low persistency. The positively moderate genetic and phenotypic correlations observed between persistency and 305DMY and TMY suggest that selecting for higher persistency may lead to the emergence of cows with increased milk yield.

**Table 3:** Genetic (below diagonal) and phenotypic correlations amongst 100DMY, 305DMY, TMY, and persistency.

**Traits**	**100DMY**	**305DMY**	**TMY**	**Persistency**
100DMY	–	0.7864	0.707	−0.2049
305DMY	0.7608	–	0.9189	0.4346
TMY	0.7196	0.9913	–	0.4182
Persistency	−0.1682	0.5028	0.5335	–

### 3.2 Genome-wide association studies

**Figure 1:**
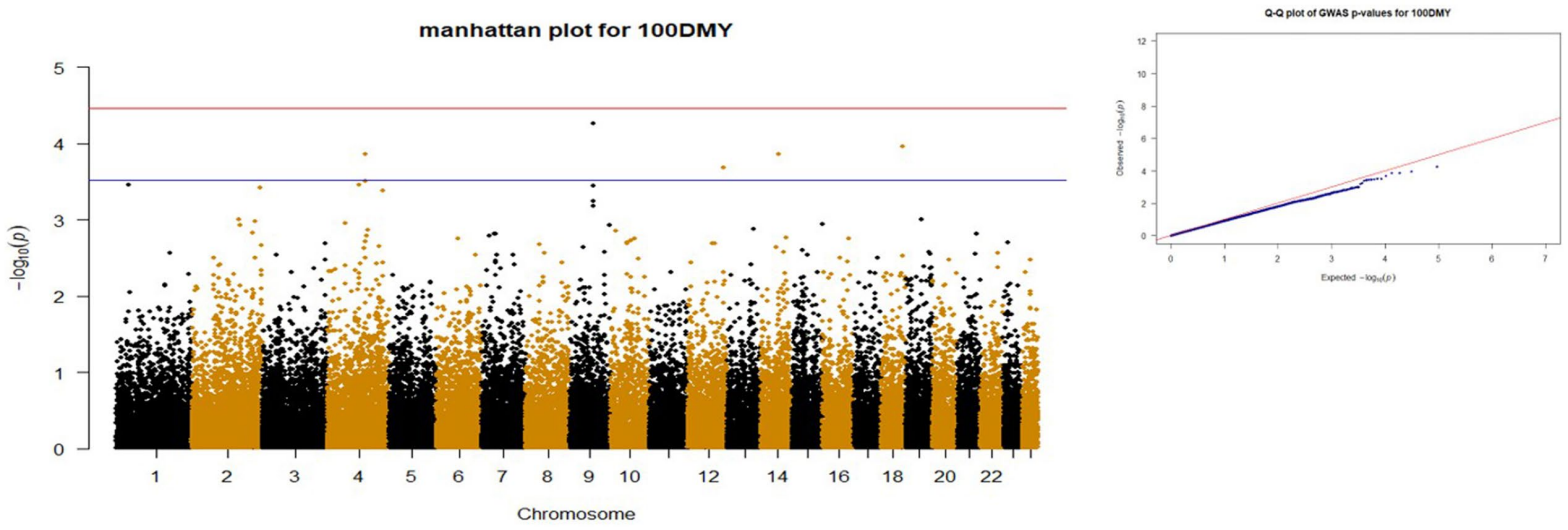
Manhattan and QQ plots of *p* values for 100DMY. The red and blue lines represents the chromosome-wide (3.42 x 10E-05) and suggestive significance (3.00 x 10E-04) thresholds, respectively.

**Figure 2:**
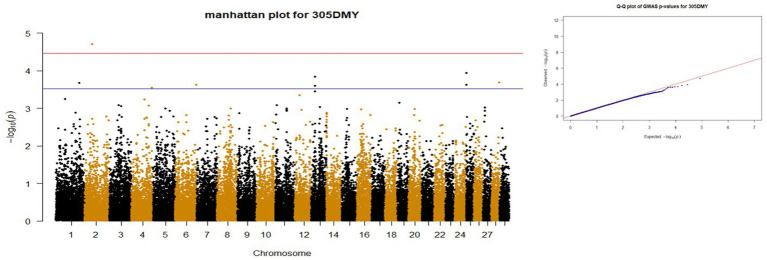
Manhattan and QQ plots of *p* values for 305DMY. The red and blue lines represents the chromosome-wide (3.42 x 10E-05) and suggestive significance (3.00 x 10E-04) thresholds, respectively.

**Figure 3:**
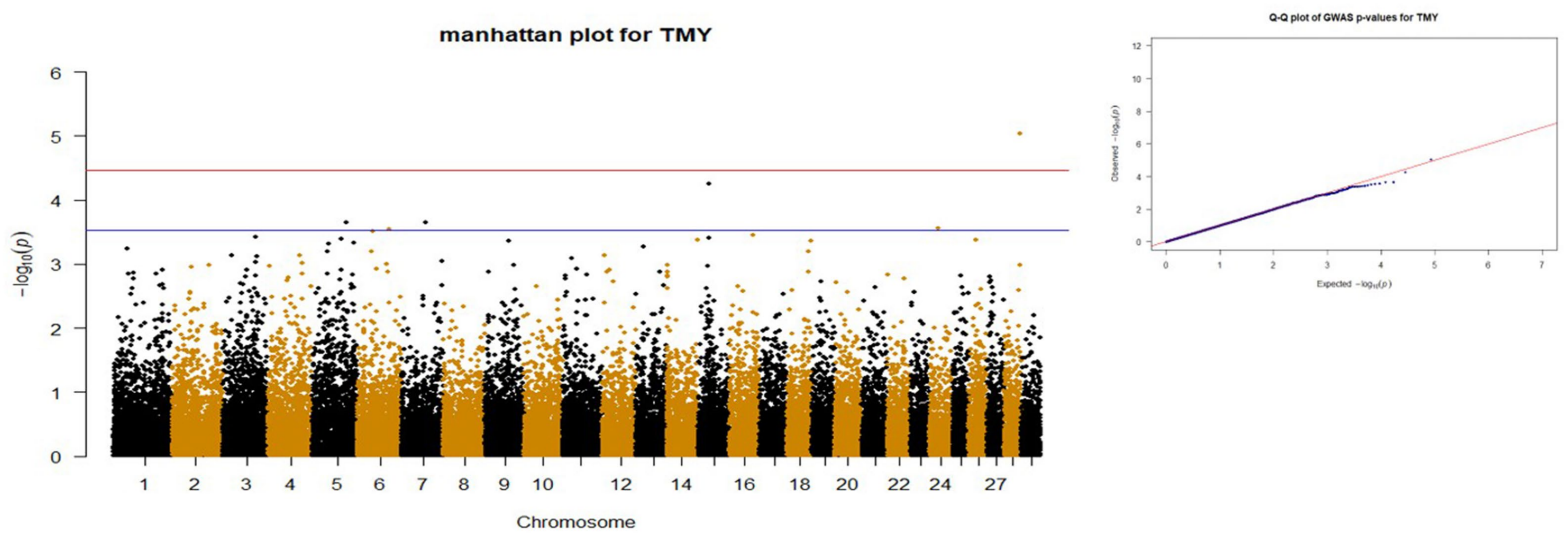
Manhattan and QQ plots of *p* values for TMY. The red and blue lines represents the chromosome-wide (3.42 x 10E-05) and suggestive significance (3.00 x 10E-04) thresholds, respectively.

**Figure 4:**
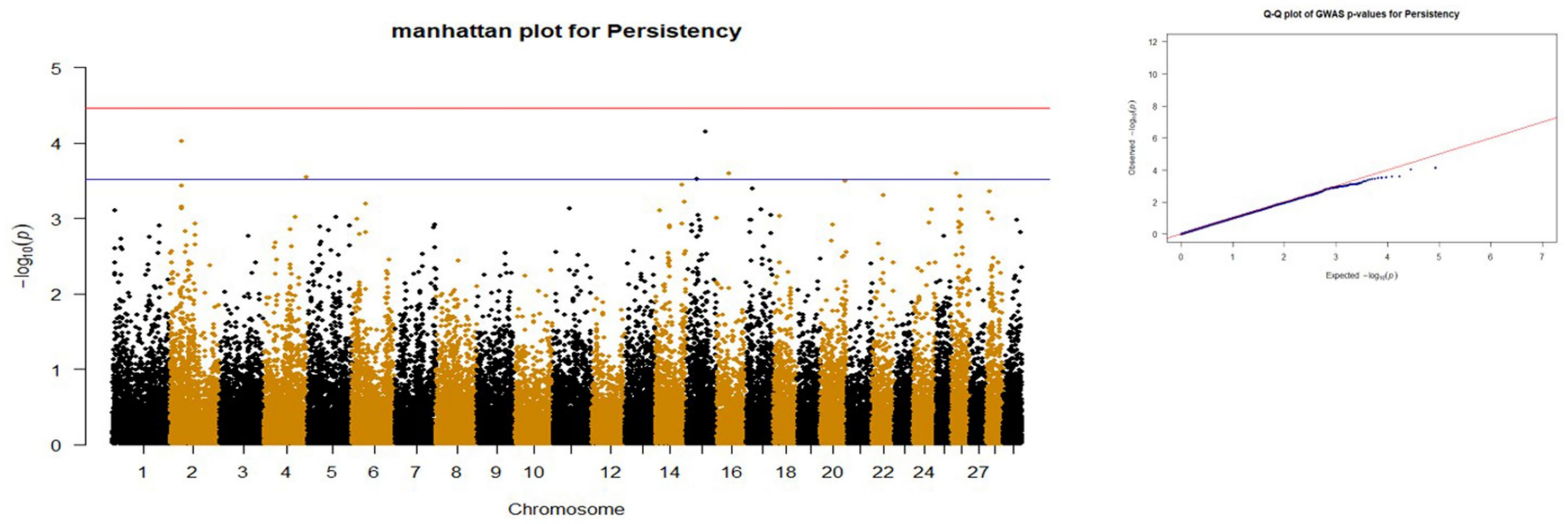
Manhattan and QQ plots of *p* values for Persistency. The red and blue lines represents the chromosome-wide (3.42 x 10E-05) and suggestive significance (3.00 x 10E-04) thresholds, respectively.

*p*-values obtained from GWAS were used to visualize the Q-Q plots. False positive results in which many SNP departed from expected probability in genome-wide association study may be due to stratification in population (7, 29). The Manhattan and Quan-tile-Quantile (Q-Q) plots illustrating the Genome-wide Association Study (GWAS) results are depicted in Figures 1–4. These plots provide visual representations of the statistical significance and distribution of genetic variants associated with the traits under investigation. Q-Q plots revealed acceptable patterns of associations and indicated that significant associated SNPs were obtained for all traits. Lambda inflation factors for 100DMY, 305DMY, TMY, and persistency were 1.02, 1.02, 0.99, and 1.01, respectively. These findings indicate the absence of notable inflations, and no corrections were per-formed for lambda since the values are very close to 1 or just slightly above. Information for the SNPs associated with studied traits were presented in Table 4. Genes within the proximity of associated SNPs were significantly enriched in some biological process (BP), molecular functions (MF), and cellular components (CC), and presented in Table 5.

**Table 4:** Significant SNPs associated with 100DMY, 305DMY, TMY, and persistency.

Traits	SNP name	SNP rs Code	Chr	Position (bp)	SNP	MAF	p-value	Nearest Gene	Distance
100DMY	ARS-BFGL-NGS-116639	rs109082401	19	22,749,859	C/G	0.069	3.22E-05	VPS53	within
100DMY	Hapmap26973-BTA-147422	rs110392985	19	12,340,926	A/G	0.382	6.45E-05	BCAS3	within
100DMY	BTA-24488-no-rs	rs41624433	13	21,236,959	A/G	0.266	1.61E-04	MALRD1	within
100DMY	BTB-00449987	rs43652357	11	1,865,280	A/G	0.184	2.00E-04	MAL	19 kb
100DMY	Hapmap51375-BTA-105537	rs41616381	20	60,783,431	T/C	0.343	2.24E-04	CTNND2	500 kb
100DMY	ARS-BFGL-NGS-113666	rs110280081	26	46,901,914	A/G	0.307	2.33E-04	DOCK1	within
100DMY	ARS-BFGL-NGS-37606	rs42295395	15	26,291,524	A/G	0.447	2.57E-04	CADM1	within
100DMY	EuroG10K_ARS-BFGL-NGS-105927	rs110983182	17	50,467,384	T/C	0.302	2.72E-04	TMEM132C	848 kb
305DMY	ARS-BFGL-NGS-20408	rs110332624	25	520,071	A/G	0.263	1.16E-04	RAB40C	within
305DMY	Hapmap42180-BTA-31841	rs41631692	13	20,011,204	T/C	0.173	1.45E-04	NRP1	within
305DMY	ARS-BFGL-NGS-71850	rs42149741	28	42,135,043	A/C	0.137	2.08E-04	GPRIN2	within
305DMY	Hapmap49636-BTA-50341	rs41639316	1	128,755,717	A/G	0.428	2.14E-04	PXYLP1	within
305DMY	ARS-BFGL-BAC-46926	rs110760798	25	406,196	T/C	0.260	2.41E-04	RAB11FIP3	within
305DMY	ARS-BFGL-NGS-106139	rs109145830	6	117,207,346	A/G	0.128	2.43E-04	LDB2	209 kb
305DMY	BTA-24488-no-rs	rs41624433	13	21,236,959	A/G	0.266	2.57E-04	MALRD1	within
305DMY	Hapmap34480-BES11_Contig223_1325	rs42760684	4	111,744,558	T/C	0.464	2.83E-04	CNTNAP2	within
TMY	ARS-BFGL-NGS-71850	rs42149741	28	42,135,043	A/C	0.137	9.26E-06	GPRIN2	within
TMY	ARS-BFGL-NGS-114922	rs43110224	15	28,966,610	A/G	0.271	5.51E-05	FXYD6	2 kb
TMY	BTA-111859-no-rs	rs41611290	5	91,044,792	T/C	0.234	2.23E-04	PLEKHA5	within
TMY	BTB-01524503	rs42644939	7	65,205,920	T/C	0.409	2.28E-04	GLRA1	94 kb
TMY	EuroG10K_BovineHD2400006223	rs137603336	24	22,794,464	T/C	0.434	2.75E-04	DTNA	within
TMY	BovineHD0600023293	rs109595562	6	84,710,597	T/C	0.395	2.87E-04	CENPC	195 kb
Persistency	ARS-BFGL-NGS-117833	rs109742720	15	55,045,620	T/G	0.383	7.18E-05	NEU3	within
Persistency	EuroG10K_ARS-BFGL-NGS-38622	rs109266162	2	29,919,184	A/G	0.397	9.43E-05	SCN7A	within
Persistency	UA-IFASA-8132	rs41626189	16	33,607,353	T/C	0.486	2.52E-04	C16H1orf100	17 kb
Persistency	ARS-BFGL-NGS-112275	rs109008551	26	14,127,433	A/G	0.154	2.54E-04	HHEX	2 kb
Persistency	Hapmap47177-BTA-111089	rs41618099	4	115,556,748	T/C	0.430	2.90E-04	KMT2C	12 kb

**Table 5:** Enrichment of gene ontology (GO) terms and functional annotations of the candidate genes included in the 200 kb upstream and downstream of the identified SNPs.

Category	Term	*P*	Genes	Fold Enrichment
MF	GO:0031720 ~ haptoglobin binding	3.1E−05	HBM, HBA, HBQ1, HBA1	63.02
BP	GO:0015671 ~ oxygen transport	3.5E−05	HBM, HBA, HBQ1, HBA1	60.74
CC	GO:0005833 ~ hemoglobin complex	4.1E−05	HBM, HBA, HBQ1, HBA1	57.85
CC	GO:0031838 ~ haptoglobin-hemoglobin complex	4.1E−05	HBM, HBA, HBQ1, HBA1	57.85
MF	GO:0043177 ~ organic acid binding	4.5E−05	HBM, HBA, HBQ1, HBA1	56.02
MF	GO:0005344 ~ oxygen carrier activity	7.3E−05	HBM, HBA, HBQ1, HBA1	48.01
MF	GO:0019825 ~ oxygen binding	1.3E−04	HBM, HBA, HBQ1, HBA1	40.33
MF	GO:0004601 ~ peroxidase activity	2.4E−04	HBM, HBA, HBQ1, HBA1	32.53
BP	GO:0042744 ~ hydrogen peroxide catabolic process	2.5E−04	HBM, HBA, HBQ1, HBA1	32.27
KW	KW-0561 ~ Oxygen transport	6.0E−04	HBM, HBA, HBA1	76.98
CC	GO:0072562 ~ blood microparticle	1.4E−03	HBM, HBA, HBQ1, HBA1	17.95
MF	GO:0008670 ~ 2,4-dienoyl-CoA reductase (NADPH) activity	1.2E−02	NME4, DECR2	168.05
MF	GO:0005515 ~ protein binding	1.5E−02	BCAS3, NHLRC4, IL10RA, ZBTB38, ARHGDIG, PLEKHA5, FBXL16, WDR24, TPBGL, FAM234A, WDR90, SLCO2B1, XRRA1, SCN9A, NPHP1, MALRD1, SPSB4	1.87
BP	GO:0006811 ~ monoatomic ion transport	2.3E−02	GLRA1, FXYD2, SLCO2B1	12.70
CC	GO:0043204 ~ perikaryon	2.4E−02	GLRA1, CTNND2, G3BP1	12.40
MF	GO:0020037 ~ heme binding	2.6E−02	HBM, HBA, HBQ1, HBA1	6.22
BP	GO:0019725 ~ cellular homeostasis	2.6E−02	RHOT2, SCN7A	73.75
BP	GO:0043269 ~ regulation of monoatomic ion transport	3.0E−02	FXYD2, FXYD6	64.53
CC	GO:0045202 ~ synapse	3.1E−02	GLRA1, RPH3AL, DTNA, CADM1, DSCAML1	4.16
BP	GO:0009062 ~ fatty acid catabolic process	3.8E−02	NME4, DECR2	51.63
BP	GO:2000649 ~ regulation of sodium ion transmembrane transporter activity	3.8E−02	FXYD2, FXYD6	51.63
MF	GO:0019911 ~ structural constituent of myelin sheath	4.2E−02	MALL, MAL	45.83
CC	GO:0009986 ~ cell surface	4.4E−02	CNTNAP2, FAM234A, MSLN, DSCAML1, PROM2	3.72

Following the genome-wide association studies, 27 SNPs were identified as potential candidates associated with 100DMY, 305DMY, TMY, and persistency in Holstein heifers (Table 4). There was a total of eight SNPs associated with 100DMY trait. One SNP exceeded the chromosome-wide significance threshold, and seven SNPs were found to be suggestively significant. The SNP rs109082401 had achieved chromosome-wide significance and is located within the VPS53 gene. Martins et al. (30) have identified this gene as pivotal for back fat thickness in Nellore cattle reared in a pasture-based system. This finding under-scores the significance of the gene in regulating adipose tissue deposition, a trait of considerable importance in cattle production systems. The second highly significant SNP (rs110392985) was detected in BCAS3 (Breast carcinoma-amplified sequence 3 homolog) gene on chromosome 19. Marina et al. (31) identified this gene as a candidate gene through a genome-wide association study investigating the genetic basis of cheese yield in Churra sheep. Parmigiano Reggiano cheese as opposed to others.

#### 3.2.1 100-day milk yield

In a study by Massender et al. (32), the BCAS3 gene was additionally linked to fat percentage in the initial lactation of Saanen goats. Similarly, Persichilli et al. (33) reported that the BCAS3 gene exhibited significance among Italian Holstein populations, particularly in those involved in the production of Also, Kim et al. (34) suggested BCAS3 gene as a candidate in a selection signature study using three Korean cattle breeds. In our study, another suggestively significant single nucleotide polymorphism (SNP), rs41624433, was identified within the MALRD1 (MAM and LDL receptor class A domain containing 1) gene. This gene has also been previously highlighted by Di Gerlando et al. (35) in their investigation focusing on milk production traits in Valle del Belice dairy sheep. Their study identified MALRD1 as a significant candidate associated with milk and fat yields, mirroring the findings observed in our research. This consistency across studies underscores the potential importance of MALRD1 in modulating milk production traits in dairy sheep populations and highlights its candidacy as a target for further investigation and breeding efforts aimed at improving milk yield and quality. This gene also reported as a potential candidate for gestation length of Large White pig population (36) and rear leg rear view of Korean Holstein cattle (37). Another suggestively significant SNP (rs41616381) is 500 kb away from the CTNND2 gene, which have been suggested to have association with milk yield for first lactation in water buffaloes (38). Gan et al. (39) reported that the CTNND2 gene was found to be related to the thyroxine hormone, which was reported to increase the milk yield, fat, and lactose content (40). Venturini et al. (41) conducted a study on Brazilian buffalo, revealing the CTNND2 gene’s association with milk, fat, and protein yields. Liu et al. (42) obtained results regarding the CTNND2 gene as a candidate for protein yield in Holsteins. Zhao et al. (43) identified this gene as linked to litter birth weight in pigs in a previous investigation. Considering the literature, this gene’s involvement in milk, fat, and protein yields suggests its potential importance in regulating milk production metabolism. Our findings are supported by an alternative SNP (rs110280081) that exceeds the suggestive threshold and is in the DOCK1 gene, which has previously been reported to be involved in milk synthesis (44, 45). We found a SNP (rs42295395) in the CADM1 gene which is assigned as a candidate gene for C18:1n9 fatty acid by Buitenhuis et al. (46). Last suggestively significant SNP for 100DMY, was in the upstream of TMEM132C gene. Interestingly, this gene has been identified in the region harboring selection signatures for Sarabi cattle in Iran, and researchers have revealed that this gene could be associated with economically important traits (47). Similarly, Yodklaew et al. (48) found that the TMEM132C gene was in the near of top 10 significant SNP markers for milk yield. In the Shanghai Holstein population, Liu et al. (42) proposed the TMEM132C gene as a potential candidate influencing protein yield.

#### 3.2.2 305-day milk yield

After the GWAS, we obtained eight suggestively significant SNPs for 305DMY. One of these SNPs (rs110332624) was in the RAB40C gene that is noted as a candidate for reproduction traits in pigs (49) and cattle (50). Gene ontology analysis revealed that the genes located around this SNP are involved in 2,4-dienoyl-CoA reductase (NADPH) activity (GO:0008670) and fatty acid catabolic process (GO:0009062). It was reported that the cows with a high liver fat content during early lactation negatively influenced the NADPH-dependent glutathione system (51). In addition, NADPH is crucial for milk fatty acid synthesis (52, 53). Genetic variations in this region may influence key pathways involved in fatty acid metabolism and redox balance, potentially impacting traits such as milk production and liver function in livestock. On the other hand, the SNP coded as rs41631692 was in the NRP1 gene, which has been previously linked with milk production and composition traits (31, 42, 54). Another SNPs, rs41639316 and rs109145830 related to the PXYLP1 and LBD2 genes that are already reported for eye muscle area of Hanwoo cattle (55) and carcass traits of Beijing-You chickens (56). The SNP rs110760798 was located within RAB11FIP3 gene. The 200 kb upstream and downstream of the locus contains several genes such as HBM, HBA, HBQ1, and HBA1 which are significantly (*p* < 0.05) enriched in two biological process named as oxygen transport (GO:0015671) and hydrogen peroxide catabolic process (GO:0042744), six molecular functions entitled as haptoglobin binding (GO:0031720), organic acid binding (GO:0043177), oxygen carrier activity (GO:0005344), oxygen binding (GO:0019825), peroxidase activity (GO:0004601), and heme binding (GO:0020037), and three cellular components called as hemoglobin complex (GO:0005833), haptoglobin-hemoglobin complex (GO:0031838), and blood microparticle (GO:0072562). Zeng et al. (57) reported that the oxygen transport of high-yielding dairy cows under heat stress has deteriorated. Moreover, blood oxygen metabolism is confirmed to be significantly connected with high production of milk in Holstein-Friesian Dairy cows (58). High haptoglobin values in dairy cows have been shown to be strongly correlated with white blood cells, indicating an inflammatory response (59). The haptoglobin concentration in dairy cattle showed a downward trend throughout lactation, and high concentrations in fresh cows could result from inflammatory illnesses without clinical disease, stress, or variations within the physiological statement (60). It is hypothesized that this region may influence oxidative stress responses and oxygen metabolism, which could subsequently impact milk production efficiency and dairy cows’ inflammatory status, particularly under conditions of heat stress and high production demands. Interestingly, another suggestively significant SNP (rs41624433) was found in the MALRD1 (MAM and LDL receptor class A domain containing 1) gene, which was also significant for 100DMY in the current study. This gene has previously explored as a candidate for milk and fat yields of Valle del Belice sheep (35), gestation length in Large White pigs (36) and rear leg rear view for Korean Holstein (37). This SNP has emerged as significant with the suggestive threshold for both 100DMY and 305DMY traits in the study. So, QTLs that significantly affect milk yield traits could be hypothesized to exist within or around the MALRD1 gene. Lastly in 305DMY, the SNP (rs42760684) located in the CNTNAP2 gene has been detected to be related with the 305DMY trait, and some literature about the CNTNAP2 revealed supportive results regarding the effects on C18:3 n3 fatty acids (61), 305-d fat yield in dairy cattle (62), scrotal circumference in Italian Simmental (63), fat yield in Murrah buffalo (64), and 305-d milk yield in Murrah buffaloes (65). So, gene could play a role in the metabolic processes related to milk production and is important for further investigations.

#### 3.2.3 Total milk yield

A total of six SNPs were identified to be associated with total milk yield (TMY) in the first lactation. The most significant marker (rs42149741) exceed the chromosome-wide threshold and is in the GPRIN2 gene on chromosome 28. According to Anton et al. (66), the GPRIN2 gene was associated with fertility in Hungarian Simmental cattle. Remarkably, Lee et al. (67) identified the GPRIN2 gene as one of the genes associated with breast cancer in humans. This underscores the potential significance of this gene in the underlying mechanism of mammary functionality. The SNP (rs43110224) located in the upstream of the FXYD6 gene with a distance of 2 kb was suggested as a candidate for TMY in Holstein cattle. This gene, in conjunction with the FXYD2 gene situated in proximity, plays a role in the biological processes of monoatomic ion transport (GO:0006811), regulation of monoatomic ion transport (GO:0043269), and regulation of sodium ion transmembrane transporter activity (GO:2000649). In another GWAS research, FXYD6 gene shows an association with Yak’s body height and researchers (68) reported that this gene encodes a transmembrane protein mediating the Na/K ion pump and accelerates the Na + deactivation and Na + pump. In alignment with our findings, Teng et al. (69) documented a significant association between the FXYD6 gene and protein percentage in Holstein cattle. This corroborates the importance of FXYD6 in regulating milk composition traits, particularly protein content, which is crucial for dairy product quality and nutritional value. Another suggestive SNP (rs41611290) is in the PLEKHA5 gene and pointed out this gene could be involved in the milk related traits. Pedrosa et al. (70) associated this gene with the fat yield of North American Holstein cattle. Furthermore, Wang et al. (9) identified the PLEKHA5 gene as being linked with both fat yield and percentage in cattle, suggesting its involvement in regulating milk composition traits. This research also reported pleiotropic effects of PLEKHA5 on various aspects of milk production, indicating that this gene may exert influence over multiple traits simultaneously. This gene has also been linked to total collagen content in Hanwoo cattle meat (71) and carcass weight in Hawai’i cattle (72). Our research indicates that the PLEKHA5 gene could be considered a potential candidate for influencing milk production in cattle. The SNP (rs42644939) located in upstream of GLRA1 gene was found to be suggestively significant for TMY in our study. In Lázaro et al.’s recent study (64), the GLRA1 gene emerged as a promising candidate influencing milk protein yield in Murrah buffaloes. This finding underscores the genetic complexity underlying the production of milk not only in cattle but also in other dairy species like buffaloes. Additionally, Makina et al. (73) and Saravanan et al. (74) both recognized this gene as a candidate in selection signature studies conducted on Holstein and Sahiwal cattle, respectively. The other suggestive SNP (rs137603336) associated with TMY of Holstein heifers is in the DTNA gene. While this gene has not been previously associated with milk yield traits, recent studies have identified it as a potential candidate for other important traits. Manca et al. (75) found evidence suggesting its involvement in residual concentrate intake in Italian Brown Swiss, while Serão et al. (76) identified it as a potential candidate for residual average daily gain in beef cattle. Moreover, Chen et al. (77) reported differential expression of the PLEKHA5 gene in cattle exhibiting high and low residual feed intake. This suggests that PLEKHA5 may play a role not only in milk production traits but also in feed efficiency, indicating its broader influence on cattle metabolism and performance. Understanding the relationship between PLEKHA5 expression and feed intake can provide valuable insights into the genetic basis of feed efficiency and guide efforts to improve feed utilization and reduce pro-duction costs in cattle farming.

#### 3.2.4 Persistency

Five SNPs were found suggestively significant in persistency. The first SNP (rs109742720) is in the NEU3 gene on chromosome 15. In a study conducted by Cochran et al. (78), the NEU3 gene was identified as being associated with several important agricultural traits in cows. These traits include daughter pregnancy rate, cow conception rate, productive life, and net merit. This research provides valuable insights into the genetic factors influencing the reproductive efficiency and overall productivity of dairy cattle. By identifying genes associated with important traits such as milk production, feed efficiency, daughter pregnancy rate, and cow conception rate, researchers acquire a more profound comprehension of the fundamental genetic mechanisms governing these traits. Such insights are essential for developing targeted breeding programs aimed at enhancing the reproductive performance and overall productivity of dairy cattle populations. By leveraging this knowledge, breeders can make informed decisions to selectively breed animals with desirable genetic traits, ultimately leading to more efficient and profitable dairy farming practices. The second SNP (rs109266162) for persistency is in the SCN7A gene on chromosome two. This gene plays a role in cellular homeostasis (GO:0019725), which is essential for maintaining equilibrium within an animal’s physiological state (79). It is particularly important for dairy cattle that produce large quantities of milk, as it helps maintain calcium (Ca) homeostasis through the absorption of Ca from the intestine and the mobilization of Ca from bone tissue (80). The SCN7A gene, which is essential for maintaining cellular homeostasis, highlights its potential involvement in calcium regulation and overall physiological balance, which are crucial for supporting high milk production and maintaining health in dairy cattle. According to findings of Rahmatalla et al. (81), the SCN7A gene may have potential associations with milk traits in cattle. This suggests a genetic link between SCN7A, and various characteristics related to milk production, which could have implications for breeding strategies aimed at enhancing milk quality and quantity in dairy cattle populations. The third SNP (rs41626189) located in C16H1orf100 gene, but no literature reports are available for the gene. Another SNP called rs109008551 was located near the HHEX gene. Pedrosa et al. (70) discovered that the SCN7A gene is linked to milk fat yield specifically in North American Holstein cattle. This suggests a significant role for SCN7A in influencing the production of milk fat in this breed, supplying valuable insights into the genetic mechanisms that dictate milk composition traits within dairy cattle populations. Mohammadi et al. (82) had suggested the HHEX gene for days open in Iranian Holstein cattle. It was determined that the gene could have effects on type 2 diabetes, insulin mechanism, and milk production (83–85) in last decades. So, Schmidt (86) previously showed that insulin injection into lactating cows interestingly caused a decrease in milk production, milk lactose content, and blood glucose levels, and an increase in milk fat and protein percentages. According to Puppin et al. (87), the HHEX gene possesses the potential to serve a critical function in the differentiation of mammary epithelial cells. This finding suggests that HHEX may contribute significantly to the complex regulatory processes involved in the function and development of mammary tissue, which is essential for milk production in dairy cattle. Understanding the role of HHEX in mammary epithelial cell differentiation could offer valuable insights into the molecular mechanisms underlying lactation and potentially inform strategies for improving milk production efficiency in dairy farming. Furthermore, the HHEX gene has been reported to be an important transcriptional regulator for miRNA regulatory networks that control milk yield (85). Therefore, the HHEX gene could be considered a candidate gene due to its possible involvement in the regulation of milk production. The last SNP (rs41618099), suggestively associated with persistency, was 12 kb away from the KTM2C gene. According to Wang et al. (88) and Wang et al. (89), this gene is associated with milk protein and calcium accumulation mechanisms. This study suggests that it may be a candidate gene for persistence.

## 4 Conclusion

This study represents the pioneering effort to detect candidate regions related with milk yield and persistency in Holstein cattle raised in Türkiye. Several SNPs were identified to be significant with these traits. Genetic parameters were estimated, revealing moderate to high heritabilities for all traits. It is noteworthy that novel candidate genes were identified, which shed light on some of the molecular pathways underlying these traits. Such discoveries significantly contribute to unravelling the genetic underpinnings of milk production traits in cattle. They offer crucial insights that could guide future breeding programs aimed at optimizing productivity and profitability within the dairy industry. By understanding the genetic factors influencing milk traits like fat yield, breeders can make informed decisions to select for desirable characteristics, ultimately leading to more efficient and economically viable dairy cattle populations.

## Data Availability

The data presented in the study are deposited in APERTA open archive repository of The Scientific and Technological Research Council of Türkiye (TÜBİTAK) with accession number 274008. https://aperta.ulakbim.gov.tr/record/274008.

## Footnotes

^1^ https://www.ncbi.nlm.nih.gov/gene/
